# The relationship between Comprehensive Geriatric Assessment parameters and depression in elderly patients

**DOI:** 10.3389/fnagi.2022.936024

**Published:** 2022-07-26

**Authors:** Yanmin Ju, Ting Liu, Kexin Zhang, Xiaoye Lin, Enlai Zheng, Jiyan Leng

**Affiliations:** Department of Cadre Ward, The First Hospital of Jilin University, Changchun, China

**Keywords:** Comprehensive Geriatric Assessment, depression, elderly patients, Geriatric Depression Scale, multivariable logistic regression analysis

## Abstract

**Background:**

Depression is common and serious among elderly patients. The treatment of elderly depression is often delayed owing to insufficient diagnosis, which eventually leads to adverse consequences.

**Aims:**

To explore the association between the parameters of the Comprehensive Geriatric Assessment and depression in elderly patients.

**Methods:**

A cross-sectional study of 211 outpatients and inpatients aged ≥ 65 years from the Comprehensive Geriatric Assessment database was conducted. A Comprehensive Geriatric Assessment inventory was prepared by compiling and screening general characteristics, chronic diseases (cardiovascular disease, diabetes, and peptic ulcer disease), nutritional status, daily living ability, anthropometric measurements (body mass index (BMI), upper arm circumference, and calf circumference), and blood biochemical indicators (hemoglobin, albumin, prealbumin, triglycerides, and low-density lipoprotein cholesterol). The Geriatric Depression Scale was also conducted for each elderly patient to screen for depression. A multivariable logistic regression analysis was used to determine the association between the parameters of the Comprehensive Geriatric Assessment and geriatric depression.

**Results:**

There were 63 patients in the depression group with a median age of 84.00 years, and 148 patients in the non-depression group with a median age of 78.50 years. After controlling for confounders, the risk of depression in elderly patients with cardiovascular diseases was 6.011 times higher than that in those without cardiovascular diseases (*p* < 0.001); and the risk of depression in elderly patients with peptic ulcer diseases was 4.352 times higher than that in those without peptic ulcer diseases (*p* < 0.001); the risk of depression in elderly patients decreased by 22.6% for each 1-point increase in the Mini Nutritional Assessment (*p* < 0.001). The risk of depression in elderly patients decreased by 19.9% for each 1-point increase in calf circumference (*p* = 0.002), and by 13.0% for each 1-point increase in albumin (*p* = 0.014).

**Conclusion:**

Our findings suggest that Comprehensive Geriatric Assessment parameters, such as cardiovascular disease, peptic ulcer disease, Mini Nutritional Assessment score, calf circumference, and albumin, were associated with depression. The Comprehensive Geriatric Assessment can assist in the early identification of depression in the elderly population.

## Introduction

Depression is a mental disorder characterized by persistent sadness, decreased interest in daily life activities, concentration difficulties, poor memory, and a lack of energy (Yeun et al., 2012). The prevalence of depression in older outpatients ranges from 10 to 20% (Beekman et al., 1999), and even higher, from 22 to 34%, in those hospitalized with somatic illnesses (Meldon et al., 1997; Raccio-Robak et al., 2002). The treatment of depression is challenging due to the complexity of the mechanisms involved. In the elderly, the responses to treatments are not as favorable as those in the younger population, thus leading to serious adverse consequences due to this condition (Liu et al., 2018; Liao et al., 2020). Moreover, depression in elderly patients is underdiagnosed, and new simple and reliable methods for the early identification of depression in these patients are urgently needed.

A Comprehensive Geriatric Assessment is a multidimensional, multidisciplinary diagnostic and therapeutic process that determines the medical, psychological, and functional capabilities of an older person; identifies at-risk patients; and may possibly guide management, treatment, and follow-up (Rubenstein et al., 1991; Fagard et al., 2017). It has been found in clinical practice that the elderly have considerable acceptability for the Comprehensive Geriatric Assessment. Previous studies have studied the potential relationship between the Comprehensive Geriatric Assessment and depression and found that the risk of depression was correlated with adverse outcomes of this assessment. Due to the medical, functional, nutritional, cognitive, and social conditions of the elderly being assessed more comprehensively, this approach is conducive to the identification of depression in the elderly (Kronfly et al., 2015).

In the present study, we hypothesized that the parameters analyzed in the Comprehensive Geriatric Assessment were related to depression in the elderly. We used the Comprehensive Geriatric Assessment to evaluate the patient’s overall situation and explore its role in identifying geriatric depression.

## Patients and methods

### Study design and participants

We conducted a cross-sectional study of 211 elderly patients in the Cadre Ward Department of The First Hospital of Jilin University from June 2020 to June 2021. All elderly patients aged ≥ 65 years with complete Comprehensive Geriatric Assessment data who agreed to participate in the study were included. Patients unable to walk due to neuromusculoskeletal disorders, such as severe cerebrovascular disease, Parkinson’s disease, knee or hip osteoarthritis, and lumbar spinal stenosis, as well as participants with severe cognitive impairment, autoimmune diseases, severe liver and kidney dysfunction, and malignant tumors, were excluded from the study. The study was conducted in accordance with the Declaration of Helsinki and approved by the Ethics Committee of The First Hospital of Jilin University.

### Measurements

#### Comprehensive Geriatric Assessment

##### General characteristics and chronic diseases

Age, gender, smoking history, drinking history, exercise, and chronic diseases (cardiovascular disease, diabetes, and peptic ulcer disease) were evaluated using a self-made face-to-face interview questionnaire.

##### Mini Nutritional Assessment

The Mini Nutritional Assessment is an 18-question nutritional assessment tool developed specifically for older adults. It consists of four components: anthropometry (body mass index (BMI), calf circumference, and arm circumference measurements), self-reported health status, dietary problems (such as, weight loss) and clinical health status. The Mini Nutritional Assessment was used to comprehensively evaluate the nutritional status of elderly patients (Guigoz et al., 1996; Guigoz, 2006). The total score of the Mini Nutritional Assessment was 30 points; if the score was < 17 points, patients were classified as having malnutrition; if the score was between 17 and 23.5 points, patients were at the risk of malnutrition; and good nutrition was reached, if the score was >23.5 points.

##### Activities of daily living and instrumental activities of daily living

Activities of daily living and instrumental activities of daily living (ADLs and IADLs) are essential for independent living and are predictors of the morbidity and mortality in older populations (Wang et al., 2020). Questions on ADLs included walking, dressing, bathing, personal hygiene, eating alone, lying down and getting up from bed and chairs, and toilet hygiene. The IADLs included preparing a hot meal, taking care of their own money, using transportation, shopping, phoning, doing light housework, and taking medicine. Participants were asked if they had difficulties with each activity and could choose to answer “yes,” “no,” “cannot,” or “not usually.” Those seniors who answered only “no” or “not usually” were classified as having no difficulties while those who answered “yes” or “cannot” for at least one of the activities were classed as having difficulties. The variables were created following the pattern of previously published studies (Nunes et al., 2019).

##### Anthropometric measurements

Height and weight were measured according to clinical standards.

Body mass index (BMI) was calculated as weight (kilogram) divided by height (meter squared).

The upper arm circumference at the level of the thickest part of the biceps brachii muscle was measured with the upper limb sags naturally (accurate to 0.1 cm).

The participants stood with their feet shoulder-width apart and the calf circumference around the most prominent part of the gastrocnemius muscle was measured (accurate to 0.1 cm).

##### Blood biochemical indicators

Blood biochemical indicators were obtained from electronic medical records, such as hemoglobin, albumin, prealbumin, triglyceride, and low-density lipoprotein cholesterol.

#### Depression assessment

The Geriatric Depression Scale screening questionnaire was designed specifically for older adults, considering that they are unable to answer too complex questions (Yesavage et al., 1982). In total, thirty items did not contain physical symptoms to avoid confusion between physical symptoms and common physical disorders in older adults (Montorio and Izal, 1996). The Geriatric Depression Scale has good reliability and validity and can be used to screen for depression in older Chinese adults, regardless of the presence of mild cognitive impairment (Huang et al., 2021). It has a cut-off score of 10 and a Geriatric Depression Scale score of >10 for the presence of depression.

## Data analysis

The SPSS/WIN 23.0 software (IBM Corp., Armonk, NY, United States) and GraphPad Prism 8 (GraphPad Prism Software Inc., San Diego, CA, United States) were used for the statistical analysis. The Kolmogorov–Smirnov test was used to test the normality of the continuous variables. Continuous variables were presented as mean ± standard deviation (SD) or median (interquartile range, IQR) based on the distribution characteristics of the data, and Student’s *t*-test or Mann–Whitney *U*-test was used to test the difference between the two groups. Classified variables were described as absolute numbers and percentages, and the difference between the two groups was tested using the chi-squared test. A multivariable logistic regression analysis was used to determine the correlation between cardiovascular diseases, peptic ulcer disease, the Mini Nutritional Assessment, calf circumference, and albumin and depression. Variables with *p* < 0.05 were selected for the multivariate analysis. The values of *p* < 0.05 were considered statistically significant.

## Results

### Baseline data of general characteristics, chronic diseases, Mini Nutritional Assessment, and activities of daily living and instrumental activities of daily living

As shown in Table 1, 211 participants participated in the study. There were 63 (29.9%) patients in the depression group with a median age of 84.00 years, and 148 (70.1%) patients in the non-depression group with a median age of 78.50 years. The difference in age between the two groups was statistically significant (*p* = 0.024). In addition, there were significant differences in the proportions of patients with cardiovascular diseases, diabetes, and peptic ulcer diseases (*p* < 0.05). The differences between the two groups in exercise, the Mini Nutritional Assessment score, and ADLs and IADLs scores were statistically significant (*p* < 0.05).

**TABLE 1:** Baseline data of general characteristics, chronic diseases, Mini Nutritional Assessment, and activities of daily living and instrumental activities of daily living (ADLs and IADLs).

Variates	Non-depression (*n* = 148, 70.1%)	Depression (*n* = 63, 29.9%)	*P*
Age, median (IQR), years	78.50 (69.00, 87.00)	84.00 (74.00, 89.00)	0.024*
Sex, male, *n* (%)	117 (55.5)	43 (20.4)	0.094
Smoke, *n* (%)	61 (28.9)	22 (10.4)	0.392
Drink, *n* (%)	68 (32.2)	21 (10.0)	0.090
Exercise, *n* (%)	98 (46.4)	29 (13.7)	0.006*
CVD, *n* (%)	50 (23.7)	48 (22.7)	<0.001*
Diabetes, *n* (%)	47 (22.3)	20 (9.5)	0.999
PUD, *n* (%)	21 (10)	26 (12.3)	<0.001*
MNA, median (IQR)	24.00 (22.00, 26.00)	19.00 (16.50, 22.00)	<0.001*
ADLs and IADLs, median (IQR)	16.00 (14.00, 23.50)	24.00 (16.50, 32.00)	<0.001*

CVD, cardiovascular disease; PUD, peptic ulcer disease; MNA, Mini Nutritional Assessment; ADLs and IADLs, Activities of daily living and instrumental activities of daily living.

*p < 0.05.

### Baseline data of the anthropometric parameters and the blood biochemical indicators

As shown in Table 2, BMI, upper arm circumference, calf circumference, and albumin were lower in the depression group than in the non-depression group (*p* < 0.05).

**TABLE 2:** Baseline data of the anthropometric parameters and the blood biochemical indicators.

Variates	Non-depression (*n* = 148, 70.1%)	Depression (*n* = 63, 29.9%)	*P*
BMI, median (IQR), kg/m^2^	24.40 (22.80, 26.2)	23.2(21.85, 24.9)	0.001*
UAC, median (IQR), cm	26.95 (25.45, 28.5)	25.90(25.15, 26.60)	0.001*
CC, mean ± SD, cm	33.6 ± 3.6	30.3 ± 2.8	<0.001*
Hemoglobin, mean ± SD, g/L	134.2 ± 19.6	129.0 ± 21.3	0.087
Alb, median (IQR), g/L	37.85 (35.40, 41.45)	35.40 (33.70, 37.10)	<0.001*
Prealbumin, median (IQR), g/L	0.22 (0.18, 0.27)	0.21 (0.19, 0.26)	0.402
Triglycerides, median (IQR), mmol/L	1.29 (0.88, 1.82)	1.08 (0.84, 1.48)	0.144
LDL-C, mean ± SD, mmol/L	2.9 ± 0.9	2.9 ± 0.9	0.766

BMI, body mass index; UAC, upper arm circumference; CC, calf circumference; Alb, albumin; LDL-C, low-density lipoprotein cholesterol.

*p < 0.05.

### Correlation between chronic diseases and Mini Nutritional Assessment and depression

**FIGURE 1:**
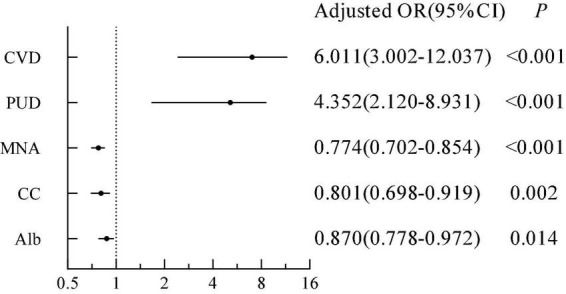
Correlation between depression and cardiovascular disease, peptic ulcer disease, Mini Nutritional Assessment, calf circumference, and albumin. CVD, cardiovascular disease; PUD, peptic ulcer disease; MNA, Mini Nutritional Assessment; CC, calf circumference; Alb, albumin.

As shown in Table 3, the results of the multivariable logistic regression model indicated that chronic diseases and Mini Nutritional Assessment scores were significantly correlated with depression (*p* < 0.05). After adjusting for age, exercise, ADLs and IADLs, patients with cardiovascular diseases were approximately six times more likely to have depression than patients without cardiovascular diseases (odds ratio (*OR*) = 6.011, 95% CI = 3.002–12.037, *p* < 0.001); patients with peptic ulcer diseases were approximately four times more likely to develop depression than those without them (*OR* = 4.352, 95% CI = 2.1201–8.931, *p* < 0.001), and the risk of depression decreased by 22.6% for each 1 point increase in the Mini Nutritional Assessment (*OR* = 0.774, 95% CI = 0.702–0.854, *p* < 0.001; Figure 1).

**TABLE 3:** Correlation between chronic diseases and Mini Nutritional Assessment and depression.

	Crude		Adjusted	
Variates	OR (95%CI)	*P*	OR (95%CI)	*P*
CVD	6.272 (3.202–12.287)	<0.001*	6.011 (3.002–12.037)	<0.001*
PUD	4.250 (2.149–8.403)	<0.001*	4.352 (2.120–8.931)	<0.001*
MNA	0.771 (0.707–0.840)	<0.001*	0.774 (0.702–0.854)	<0.001*

CVD, cardiovascular disease; PUD, peptic ulcer disease; MNA, Mini Nutritional Assessment; OR, odds ratios; Adjusted for age, exercise, ADLs and IADLs.

*p < 0.05.

### Correlation between body mass index, upper arm circumference, calf circumference, and albumin and depression

As shown in Table 4, the results of the multivariable logistic regression model indicated that BMI, upper arm circumference, calf circumference, and albumin were significantly correlated with depression (*p* < 0.05). After adjusting for age, exercise, cardiovascular diseases, peptic ulcer diseases, ADLs and IADLs, and Mini Nutritional Assessment, the risk of depression decreased by 19.9% for each 1-point increase in calf circumference (*OR* = 0.801, 95% CI = 0.698–0.919, *p* = 0.002), 13.0% for each 1- point increase in albumin (*OR* = 0.870, 95% CI = 0.778–0.972, *p* = 0.014; Figure 1).

**TABLE 4:** Correlation between body mass index, upper arm circumference, calf circumference, and albumin and depression.

	Crude		Adjusted	
Variates	OR (95%CI)	*P*	OR (95%CI)	*P*
BMI (kg/m^2^)	0.852 (0.761–0.953)	0.005*	0.942 (0.816–1.087)	0.413
UAC (cm)	0.832 (0.737–0.939)	0.003*	0.974 (0.825–1.150)	0.755
CC (cm)	0.742 (0.668–0.827)	<0.001*	0.801 (0.698–0.919)	0.002*
Alb (g/L)	0.837 (0.768–0.912)	<0.001*	0.870 (0.778–0.972)	0.014*

BMI, body mass index; UAC, upper arm circumference; CC, calf circumference; Alb, albumin; OR, odds ratios; Adjusted for age, exercise, cardiovascular diseases, peptic ulcer diseases, ADLs and IADLs, Mini Nutritional Assessment.

*p < 0.05.

## Discussion

Our study is the first to report an association between Comprehensive Geriatric Assessment parameters and depression in elderly patients. In analyzing this relationship, we found some indicators that were closely related to depression. In particular, our results show that cardiovascular disease, peptic ulcer disease, Mini Nutritional Assessment and nutrition-related indicators of calf circumference, and albumin were associated with depression in senile patients.

Depression significantly increases the risk of cardiovascular disease in older adults, according to a recent prospective cohort study (Yu et al., 2022). In addition, a study of people with dementia found that the risk of malnutrition was significantly associated with muscle function and the risk of sarcopenia, frailty, depression, as well as reduced quality of life in people with dementia (Chou et al., 2022). Moreover, the results of a large cross-sectional study of malnutrition in older patients with obesity using the Comprehensive Geriatric Assessment suggested that malnutrition in patients with obesity is associated with depression (Soysal et al., 2022). These latest findings demonstrate the association in older patients across different conditions, between depression and both cardiovascular disease and malnutrition, which involve many factors in the Comprehensive Geriatric Assessment. However, the present study systematically analyzed the association of factors included in the Comprehensive Geriatric Assessment with depression in older adults. In addition to the indicators described in the above studies, our research also included blood biochemical indicators, such as albumin, low-density lipoprotein, and others, and thus broadly explored the correlation between the Comprehensive Geriatric Assessment and depression. This provides a basis of evidence that allows the early identification of depression in older adults.

The study group consisted of 7,589 patients from 24 European countries who were hospitalized for a coronary heart disease event and were followed up for 1.4 years. The results showed that 22.4% of patients suffered from depression, that is, cardiovascular disease is associated with an increased risk of depression (Pogosova et al., 2017). Another study of people aged 60 years or older in the United States showed that depression and its symptoms in older age are associated with an increased risk of cardiovascular mortality, which may direct future research toward treating advanced depression as a means of reducing mortality (Wei et al., 2022). A recent review, concluded that individuals with depression had significantly increased markers of inflammation, which caused a weakened structure of the arteries. This resulted in an acceleration of atherosclerosis, leading to cardiovascular events (Malik et al., 2021). Thus, there is an evident correlation between cardiovascular disease and depression.

Domestic and foreign scholars have found that peptic ulcer disease is correlated with depression in the elderly. A large-sample study in Korea showed that depression was associated with an increased risk of peptic ulcer disease. Conversely, peptic ulcer disease has been associated with an increased risk of depression. These associations were maintained in most age and sex subgroups, indicating that depression and peptic ulcers had reciprocal associations (Kim et al., 2020). In addition, a 36-month follow-up study of 2,850 elderly patients living alone in eight Grade A hospitals in China found that the cumulative incidence of peptic ulcer disease in patients with depression was higher than that in patients without depression, and that baseline depression was associated with a subsequent incidence of peptic ulcer disease (Fang et al., 2019).

More importantly, however, we found a significant association between depression and Mini Nutritional Assessment, calf circumference, and albumin, which have previously been shown to reflect nutrition levels in elderly hospitalized patients. Due to the aging of the world’s population, scholars from all areas of the society are increasingly concerned about nutrition and depression in the elderly population. A related study published last year on *Nutritions* found a strong correlation between nutritional status and depression (McFarland et al., 2020). Similar studies have shown that the Mini Nutritional Assessment scores of elderly patients with depression were significantly lower than those of patients without depression, suggesting a correlation between depression and malnutrition (German et al., 2008). Among them, calf circumference is an important indicator of Mini Nutritional Assessment, and there is a significant correlation between calf circumference and the Geriatric Depression Scale in the elderly. The smaller the calf circumference is, the lower the Mini Nutritional Assessment score is (McFarland et al., 2020). Another measure of albumin, the most abundant protein in the body, is an important indicator of a patient’s nutritional status. Small changes in albumin that correspond to depression actually reflect larger changes in nutrition levels, and albumin changes either cause depressive symptoms, which is a very important biological marker. In terms of the overall biological changes corresponding to depression, the changes in albumin are relatively larger, which have important implications related to the relationship between biological factors and mental states (McFarland et al., 2020). As mentioned above, the prevalence of depression in the elderly is gradually increasing, and depression is related to a variety of diseases, and has different degrees of risk for the patients’ health; therefore, its early identification and prevention has become crucial. The above comprehensive results related to depression in the elderly can be used to assist in the identification of depression in elderly, and provide a basis for diagnosis and further treatment, and generally apply to the elderly.

This study has limitations that are worth considering. First, based on studies that on one hand suggested that gene conversion may be an effective treatment for cerebral ischemia (Driga et al., 2021), and on the other hand investigated PON1, PON2, PON3, and MPO gene expression in patients with depression (Blizniewska-Kowalska et al., 2022), we considered that elderly patients with depression can also undergo genetic testing, which may provide information for the prediction of depression. In the future, it is worthwhile to collect a larger sample and add a gene marker project to explore the genetic characteristics of depression in the elderly and develop tools for its prediction. Second, non-medical workers in the community cannot interpret the results of the Comprehensive Geriatric Assessment, which affects the early prediction and intervention of depression by non-medical workers in the community. Third, due to the difference in the medical standards of different medical institutions and the patients’ understanding of their own previous health conditions, the integrity and credibility of the Comprehensive Geriatric Assessment are reduced, thus affecting the evaluation results. Finally, although our Comprehensive Geriatric Assessment comprehensively assessed the overall situation of the elderly, intelligence and frailty related tests and scales, such as gait speed and grip strength were not included in this experiment. The content of the Comprehensive Geriatric Assessment should be further improved in future studies on the elderly, such as adding the Mini-Mental State Examination, to clarify the correlation between mild, moderate, and severe cognition and depression. This a cross-sectional study conducted in a cadre ward, therefore, our findings may not be directly extrapolated to older people in the community.

## Conclusion

Our findings suggest that Comprehensive Geriatric Assessment parameters, such as cardiovascular disease, peptic ulcer disease, the Mini Nutritional Assessment score, calf circumference, and albumin were associated with depression. The Comprehensive Geriatric Assessment can assist in the early identification of depression in the elderly population.

## Data Availability

The raw data supporting the conclusions of this article will be made available by the authors, without undue reservation.
